# Fight Fungi with Fungi: Antifungal Properties of the Amphibian Mycobiome

**DOI:** 10.3389/fmicb.2017.02494

**Published:** 2017-12-14

**Authors:** Patrick J. Kearns, Sarah Fischer, Saioa Fernández-Beaskoetxea, Caitlin R. Gabor, Jaime Bosch, Jennifer L. Bowen, Michael F. Tlusty, Douglas C. Woodhams

**Affiliations:** ^1^Department of Marine and Environmental Sciences, Northeastern University, Nahant, MA, United States; ^2^Department of Biology, University of Massachusetts Boston, Boston, MA, United States; ^3^Museo Nacional de Ciencias Naturales, Consejo Superior de Investigaciones Científicas, Madrid, Spain; ^4^Department of Biology, Population and Conservation Biology Program, Texas State University, San Marcos, TX, United States; ^5^Anderson Cabot Center for Ocean Life, New England Aquarium, Boston, MA, United States; ^6^School for the Environment, University of Massachusetts Boston, Boston, MA, United States

**Keywords:** disease ecology, mycobiome, microbiome, chytrid, ITS, 16S rRNA, amphibian, fungal disease

## Abstract

Emerging infectious diseases caused by fungal taxa are increasing and are placing a substantial burden on economies and ecosystems worldwide. Of the emerging fungal diseases, chytridomycosis caused by the fungus *Batrachochytrium dendrobatidis* (hereafter *Bd*) is linked to global amphibian declines. Amphibians have innate immunity, as well as additional resistance through cutaneous microbial communities. Despite the targeting of bacteria as potential probiotics, the role of fungi in the protection against *Bd* infection in unknown. We used a four-part approach, including high-throughput sequencing of bacterial and fungal communities, cultivation of fungi, *Bd* challenge assays, and experimental additions of probiotic to Midwife Toads (*Altyes obstetricans*), to examine the overlapping roles of bacterial and fungal microbiota in pathogen defense in captive bred poison arrow frogs (*Dendrobates* sp.). Our results revealed that cutaneous fungal taxa differed from environmental microbiota across three species and a subspecies of *Dendrobates* spp. frogs. Cultivation of host-associated and environmental fungi realved numerous taxa with the ability to inhibit or facilitate the growth of *Bd*. The abundance of cutaneous fungi contributed more to *Bd* defense (~45% of the fungal community), than did bacteria (~10%) and frog species harbored distinct inhibitory communities that were distinct from the environment. Further, we demonstrated that a fungal probiotic therapy did not induce an endocrine-immune reaction, in contrast to bacterial probiotics that stressed amphibian hosts and suppressed antimicrobial peptide responses, limiting their long-term colonization potential. Our results suggest that probiotic strategies against amphibian fungal pathogens should, in addition to bacterial probiotics, focus on host-associated and environmental fungi such as *Penicillium* and members of the families Chaetomiaceae and Lasiosphaeriaceae.

## Introduction

Amphibians are globally at risk from a range of factors, not least of which are emerging fungal pathogens (Wake and Vrendenburg, 2008; Fisher et al., 2012). The chytridiomycete fungus, *Batrachochytrium dendrobatidis* (hereafter *Bd*), induces a skin infection in amphibians that disrupts osmotic balance, leading to mortality (Voyles et al., 2009), and has been linked to the extinction or population decline of numerous amphibian species. While chytridiomycosis often has a high mortality rate, species and populations can exhibit variable resistance to *Bd* infection and this differential susceptibility is due in part to skin microbiota (Woodhams et al., 2014).

The microbiome, or the micro-organisms that live on and in organisms, can affect host health by influencing development, behavior, metabolism, and inflammation response (Cho and Blaser, 2012). In amphibians, the bacterial populations associated with the skin can provide protection against *Bd* infection through the production of anti-fungal metabolites and can be considered part of the amphibian's immune system (Bletz et al., 2013). While fungi have been known to produce anti-microbial compounds for some time (e.g., *Penicillin*, Strobel and Daisy, 2003), their effects on amphibian health and interactions with host immune defense are not well known. In particular, studies of cutaneous non-*Bd* fungal communities on amphibians infected with *Bd* are lacking, despite a growing literature on bacteria that are often suggested for probiotic applications (Rebollar et al., 2016; Woodhams et al., 2016).

Fungi are diverse, and play prominent roles in ecosystems as decomposers, pathogens, and parasites (Kirk et al., 2008; Fierer, 2017). In addition, fungi form intricate symbioses with plants, aiding them in host defense and nutrient acquisition (Philippot et al., 2013). The role of bacteria in non-plant host-associated systems has received substantial attention, however the role fungi play in animal hosts is unclear (Huffnagle and Noverr, 2013). Non-*Bd* fungi have been detected on amphibians (Gugnani et al., 1980; Czeczuga et al., 1998) and other non-*Bd* fungi have been shown to cause disease in amphibians (e.g., Frank, 1976; Wright and Whitaker, 2001). Despite the abundance of information about *Bd*-host interactions, we lack an understanding of the diversity and metabolic capabilities of non-pathogenic fungi associated with amphibians.

Because of the efficacy of fungi at inhibiting other infectious diseases (Fox, 2015) and the potential role microbes can play in the host immune response (Cho and Blaser, 2012), we tested the hypothesis that that cutaneous fungal taxa supplement host defense against chytridiomycosis. Further we hypothesized that *Bd* inhibitory fungal taxa would comprise a significantly greater proportion (as percentage of reads) of the cutaneous microbiome than bacteria on three species and one subspecies of poison arrow frogs (*Dendrobates* spp.). To test these hypotheses we partnered with the New England Aquarium to examine the microbiota of captive poison arrow frogs including *Dendrobates auratus, D. leucomelas*, and two subspecies of *D. tinctorius* as well as their tank environment using high-throughput sequencing of bacterial and fungal communities and cultivation of fungal taxa. In 2008, the Aquarium's *Dendrobates* collection experienced a *Bd* infection that eliminated populations of *D. auratus* and *D. tinctorius* while *D. leucomelas* individuals survived this exposure (Hirokawa et al., 2008). The controlled rearing conditions of these frogs on exhibit at the New England Aquarium, coupled with their divergent history of response to *Bd* infection, provides an excellent system to examine the relative contributions of bacterial and fungal skin communities on differential host defense. In addition, we tested whether fungal or bacterial probiotics can elicit a corticosterone stress response and examined whether skin peptide defense capacity was altered by the treatment.

## Materials and methods

### Sample collection

Frogs were housed on exhibit at the New England Aquarium (Boston, MA, USA) and in its related holding facility 12 km south in Quincy, MA (Table 1). All frog species on exhibit at the New England Aquarium were housed in a single tank. This 3 m^3^ display had a glass public facing front and a solid fiberglass housing with an 80 L sump for water reserve that was pumped up to the exhibit. Water and biofilm samples were collected from the sump. The frogs at the Quincy holding facility were housed in one of four species-specific plexiglass holding cages. In 2015, holding cages had a paper-towel on the floor and the cage was tilted so there was a 3-cm deep water pool along one edge. In 2016, the holding cages were enhanced with soil, moss, and other plants, and had a 4-cm deep pool integrated into the enclosure. Each cage held 5 to 8 frogs and the closest five frogs to the door were sampled to avoid inducing stress from “chasing.” In these holding cages, biofilms were collected from the edge of the water pool. Approximately 75% of the frogs of each species in collection were bred at the Quincy holding facility, with the remainder originating in other Association of Zoo and Aquarium accredited institutions.

**Table 1:** Table of the number of amphibians and environmental samples collected in 2015 and 2016 from the two locations (Boston and Quincy, MA, USA) sampled.

	**2015- Quincy**	**2016- Quincy**	**2016- Boston**
*D. leucomelas*	5	5	1
*D. auratus*	5	5	3
*D. tinctorious* “azureus”	4	4	3
*D. tinctorious*	Not sampled	5	6
Water	5	5	2
Tank biofilm	5	5	4

Frogs were individually selected, rinsed with 15-mL of sterile water to remove transitory microbes, and swabbed on the ventral surface (Table 1) with *fine-tipped rayon swabs (Molecular Wire and Equipment MW113)*. Swabs were placed in cryovials and stored on dry ice for transportation to the lab. All samples were kept at −80°C until DNA extraction. Water was collected in 15 mL sterile tubes, stored on ice, and filtered at University of Massachusetts, Boston through 0.2 μm sterivex filters to capture bacteria and fungi. Biofilms were also swabbed from the tank surface and stored frozen in sterile cryovials.

### DNA extraction, PCR, and sequencing

DNA was extracted from frog and tank biofilm swabs using the MoBio PowerSoil Total DNA Isolation kit (Carlsbad, CA, USA) following manufacturer's instructions. Water samples were extracted using the MoBio PowerWater Total DNA Isolation kit following manufacturer's instructions. DNA extractions were verified by gel electrophoresis and when bands weren't visible, samples were checked with PCR using general bacterial primers (Caporaso et al., 2012). Samples for bacterial community analysis were amplified in triplicate using the primer pair 515F and 806R (Caporaso et al., 2010) following previously published conditions (Caporaso et al., 2012). Primer constructs had Illumina adaptors and 12-bp GoLay barcodes. Proper product formation was verified with gel electrophoresis and samples were purified with the Qiagen QiaQuick Gel Purification Kit (Qiagen, Valencia, CA). DNA from each sample was quantified with a Qubit fluorometer (ThermoFisher, Waltham, MA, USA) and pooled in equal masses for paired-end 151-bp sequencing on an Ilumina MiSeq using V2 chemistry at the University of Massachusetts Boston.

Fungal communities were amplified in triplicate with primers ITS1F and ITS2R (Walters et al., 2016) targeting the fungal internal transcribed spacer region (ITS). We used primer constructs that had overhang sequences to allow downstream addition of dual Illumina indices and adapters. Proper product formation was verified with gel electrophoresis and samples were purified with the Qiagen QiaQuick Gel Purification kit. A second 8-cycle PCR was performed with the Illumina Nextera XT2 kit following the manufacturer's instructions to ligate dual indices and Illumina adaptors to each sample. Amplified DNA from each sample were purified using the Qiagen PCR Purification kit, quantified with a Qubit, and pooled in equal mass for paired-end 151-bp sequencing on an Illumina MiSeq using V2 chemistry (Caporaso et al., 2011).

### Sequence and statistical analyses

Paired-end reads from 16S rRNA gene or ITS gene sequences were first joined with fastq-join (Aronesty, 2011) and then quality filtered and demultiplexed in QIIME (version 1.91; Caporaso et al., 2010) following previously published guidelines (Bokulich et al., 2013). Fungal reads were further quality filtered using ITSx to remove 5S and 18S fragments, which improves fungal analyses (Bengtsson-Palme et al., 2013). Both bacterial and fungal sequences were clustered into operational taxonomic units (OTUs) at 97% sequence identity using uClust (Edgar, 2010) against the GreenGenes (version 13.5) and UNITE (version 7.0) databases respectively. Following clustering, OTUs appearing only once (singeltons) and OTUs matching archaea, chloroplasts, and protists were removed from both datasets. Beta diversity was calculated using Bray-Curtis similarity on OTU tables normalized to the lowest sampling depth (9,563 for bacteria and 6,753 for fungi). Beta diversity was visualized with a principal coordinates analysis. Due to the concerns with rarefaction (e.g., McMurdie and Holmes, 2014), we calculated beta diversity on unrarefied OTU tables and compared the distance matrix to a rarefied distance matrix using a mantel test. The mantel test revealed no significant difference between rarefied and unrarefied distance matricies, thus we used rarefied data for subsequent analyses. Significant differences in community composition were assessed with a permutational multivariate analysis of variance with 10,000 permutations in QIIME (Anderson, 2001). We included habitat (frog species, water, biofilm) as variables in the overall PERMANOVA, and used additional PERMANOVAs to assess all pair-wise comparisons.

### Quantitative PCR

Quantitative PCR (qPCR) was performed to assess the copy number of the 16S rRNA gene and fungal ITS region. DNA and standards were first quantified with a Qubit fluorometer (ThermoFisher, Waltham, MA, USA). All samples were normalized to 3 ng μL^−1^ and serial dilutions of standards were prepared from purified PCR product of each gene. DNA from each sample was amplified in triplicate, along with standards and internal controls on a Strategene MX-3005P quantitative thermal cycler (Stratagene, La Jolla, CA, USA). 16S rRNA and ITS genes were amplified in 25 μL reactions using 0.25 μL of each primer, 12.5 μL of Qiagen QuantiTect SYBR Green PCR Master Mix, 1 μL of DNA template, and 11 μL PCR grade water. Bacterial qPCR was performed with primers 357F and 515R (Biddle et al., 2008) following conditions described by Bowen et al. (2011). Fungal qPCR was performed with primers ITS1F and ITS2R with the same cycling conditions as the 16S rRNA gene. Proper product formation was verified with melt curves and gel electrophoresis. All standard curves possessed a high degree of linearity (>0.99 *R*^2^) and PCR efficiency ranged from 95 to 101% for both bacterial and fungal qPCR. To assess differences in the abundance of 16S rRNA gene and ITS copy number among frog species and their environment we used an ANOVA in R (R Core Team, 2012) with a Tukey HSD test for multiple comparisons. We ensured data met the assumptions of an ANOVA including assessing variance with a Bartlett test.

### Fungal isolation and *Bd* assays

To isolate fungi we swabbed the tank biofilm and the ventral surface of frogs in 2015 and plated the swabs on Potato Dextrose Agar and Sabouraud Dextrose Agar. One milliliter of water was spread on Potato Dextrose Agar and Sabouraud Dextrose Agar as well. Plates were incubated at 25°C in the dark for 3 days and all visually distinct (shape and color) isolates were picked and isolated on Potato Dextrose Agar. Isolates were identified with sequencing at the Massachusetts General Hospital DNA Core Facility using the primer pair ITS1F and ITS4R (White et al., 1990). We clustered our isolates into OTUs at 97% identity as described above and assigned taxonomy in QIIME with BLASTn and the UNITE database. We defined a taxonomic hit with a *e*-value < 1e^−5^ and a percent identity of 97% or greater.

To test the efficacy of isolates for inhibition of *Bd* zoospore growth we followed a protocol outlined previously (Woodhams et al., 2014). Briefly, all isolates were grown in 1% tryptone broth overnight in sterile centrifuge tubes at room temperature and growth was verified by checking for turbidity. Following confirmation of growth, samples were centrifuged at 2,225 × g for 5 min to pellet the cells and the liquid was filtered through 0.22 μm filters. Isolate filtrates were kept at −20°C until needed for growth inhibition assays. Two strains of *Bd* from the Global Panzootic Hypervirulent lineage (JEL 197 and 423) were grown on 1% tryptone agar for 4–7 days to allow for the production of zoospores. Plates were flooded with 1% tryptone and the liquid was filtered through 0.45 μm filters. *Bd* zoospores were counted on a haemocytometer and diluted to 5 × 10^6^ zoospores mL^−1^. To assay the inhibition of *Bd* we inoculated 96-well plates with 50 μL *Bd* zoospores and 50 μL isolate filtrates. For negative controls we used heat killed *Bd* and wells containing no zoospores and for positive controls 50 μL of tryptone was added to zoospores. Growth was measured as changes in optical density at 480 nm at days 0, 3, 5, and 7. Percent growth of *Bd* in the presence of metabolites was calculated by taking the slope of the optical density over the 7-day incubation, subtracting the optical density of the negative controls, and dividing by the average slope of the growth in the positive control wells. Differences in growth between isolates and controls was measured with a *t*-test in R (R Core Team, 2012) using a Benjamini-Hochberg correction for multiple comparisons. To determine the phylogenetic relationship among fungal taxa we aligned ITS sequences with clustalW (Thompson et al., 2003) and constructed a phylogenetic tree based on maximum likelihood using RAxML (Stamatakis, 2014) and visualized the tree with the Interactive Tree of Life (Letunic and Bork, 2007). We tested confidence in tree topology using bootstrapping with 1,000 restarts. To test for a phylogentic signal of *Bd*-growth inhibition or facilitation within our isolates, we used a UniFrac significance test (Lozupone et al., 2006) and a phylogenetic signal analysis in the R package picante (Kembel et al., 2010).

#### The abundance of *Bd* inhibitory/facilitating microbes

To assess the proportion of reads in the bacterial communities that were inhibitory toward *Bd* we filtered our data set against a database of known *Bd*-inhibiting bacteria (Woodhams et al., 2015) in QIIME and filtered our fungal dataset against our *Bd* inhibiting/facilitating isolates. To do this, we performed a closed reference OTU pick against the GreenGenes and UNITE databases and filtered OTUs matching inhibitory/facilitating taxa from our dataset and compared the number of reads before and after filtering. We assessed significant differences among fungi and bacteria as well as among frog species with an ANOVA followed by a Tukey HSD test for pair-wise comparisons. We visualized the results using a heatmap and dendrograms to assess similarity of *Bd*-inhibitory communities. To test for differential abundance of OTUs between frog species and the environment, we used a Kruskal-Wallis test in R. Dendrograms were calculated using the weighted pair group method and arithmetic mean (WPGMA) clustering and significant differences among communities (between frog species and between frog species and their environment) were tested with a PERMANOVA. We tested for differences in percentages of inhibitory taxa between frogs and the environment using an ANOVA with a Tukey HSD test for multiple comparisons. We used a bipartite network analysis to determine the interaction of inhibitory and facilitating bacterial and fungal taxa using a previously described method (Bowen et al., 2013). This network determines positive association (presence of OTUs) of OTUs to a given environment. We included sample types as their own nodes using the Fruchterman and Reingold (1991) algorithm for ease of visualization. Networks were visualized using the R package network (Butts et al., 2012).

### Testing amphibian immune and stress response to bacteria and fungi

Midwife toads, *Alytes obstetricans* (*n* = 29), were raised in captivity from larvae at the Breeding Centre of Endangered Amphibians of the Guadarrama Mountains in Spain. Toad research conformed to the legal requirements of Consejerias de Medio Ambiente of Madrid. The toads were maintained on a 12:12 h light cycle and fed *Acheta domesticus ad libitum*. After experimental treatments described below, toads were released into an outdoor mesocosm containing natural vegetation, a small pond, and pile of rocks for shelter. Each toad was photographed for individual identification upon recapture based on unique markings.

Midwife toads were randomly assigned to one of four treatments. Control toads (*n* = 12) were bathed in 20 ml sterile water for 1 h. Toads treated with probiotics were bathed in 20 ml water containing 1 ml of either *Penicillium expansum* (*n* = 9), *Janthinobacterium lividum* (isolate 77.5b1, 56 × 10^7^ CFU, *n* = 4), or *Flavobacterium johnsoniae* (isolate 70c, 19 × 10^7^ CFU, *n* = 4) for 1 h. The *P. expansum* was grown on Sabouraud Dextrose agar, while freshly growing bacteria were rinsed directly from 15 mm Petri plates with R2A agar media supplemented with 1% tryptone. Probiotic isolates were originally collected from wild *A. obstetricans* near Basel, Switzerland and chosen for this experiment based on their ability to inhibit *B. dendrobatidis* growth (Woodhams et al., 2014). Isolates used in this study were deposited in the Culture Collection of Switzerland (CCOS 423 & 433, http://www.ccos.ch/).

We assessed the stress response of frogs to probiotics using water-borne corticosterone release rates. Corticosterone is the primary amphibian stress hormone and water-borne corticosterone release rates are highly correlated with circulating corticosterone levels measured from plasma (Gabor et al., 2013). An hour after removing the toads from probiotic treatments, they were placed in 40 ml of sterile water within a 100 ml beaker for 1 h to collect water-borne hormones. Frogs were carefully lifted out of the beaker and the remaining water sample was saved to assay corticosterone release rates. Water samples were immediately frozen at −20°C and the hormones were extracted from the thawed water using C18 solid phase extraction columns (SepPak Vac 3 cc / 500 mg; Waters, Inc., Milford, MA, USA) with Tygon tubing (Saint Bobain formulation 2475) under vacuum pressure. After extraction the columns were immediately frozen at −20°C and sent to Texas State University where they were eluted with methanol and then evaporated with nitrogen gas following Gabor et al. (2013). The residue was then resuspended in 5% ethanol and 95% EIA buffer (provided by Cayman Chemicals Inc. Ann Arbor, MI, USA) for a final re-suspension volume of 250 μL. Corticosterone release rates were measured in duplicate for all samples with an enzyme-immunoassay (EIA) kit (Cayman Chemicals Inc.) on a fluorescent plate reader (BioTek Powerwave XS). We examined the differences among treatment groups in the initial corticosterone stress (natural log transformed) using ANOVA with Tukey HSD pairwise comparisons (R Core Team, 2012), ensuring data met the assumptions of an ANOVA, including assessing variance with a Bartlett test.

Following the hormone assay we released toads into the same outdoor mesocosm for 4 weeks at which point toads were recaptured and sampled to measure their skin peptide defense capacity according to established methods (Woodhams et al., 2014). Peptide quantities recovered were compared among treatment groups. Peptides at a concentration of 500 μg ml^−1^ were tested for ability to inhibit the growth of *B. dendrobatidis, J. lividum*, and *F. johnsoniae*. The differences among treatment groups were compared using ANOVA with Tukey HSD pairwise comparisons (R Core Team, 2012), ensuring all assumptions of an ANOVA were met.

### Ethics statement

Experiments at the New England Aquarium were conducted under the supervision of trained veterinary staff and were conducted in accordance with the New England Aquarium Animal Care and Use Committee Proposal 2015-01. Experiments with midwife toads were conducted under permit number 10/032921.9/12 from Conseneria de Medio Ambiente of Comunidad de Madrid.

## Results

### Community composition and abundance

**Figure 1:**
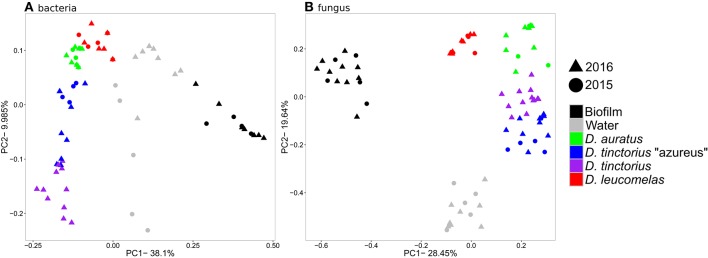
Principal coordinates analysis based on Bray-Curtis similarity for the bacterial **(A)** and fungal **(B)** communities from *Dendrobates* spp. frogs and their environment. The data for 2016 include frogs from both single species and mixed species enclosures.

In the winter of 2015 and 2016 we sampled skin microbiomes from poison arrow frogs both from a mixed-species exhibit at the New England Aquarium (Boston, MA, USA), as well as from single species holding (Animal care facility, Quincy MA, USA). High-throughput sequencing of the 16S rRNA gene and fungal intergenic transcribed spacer (ITS) region revealed distinct communities of microbes associated with host skin compared to the microbial communities found in the frog's environment [Figure 1; PERMANOVA, Bacteria: *p* < 0.001, *F*_(5, 62)_ = 7.89, all pair-wise *p* < 0.01; Fungi: *p* < 0.01, *F*_(5, 45)_ = 29.43, all-pairwise *p* < 0.001]. Furthermore, we identified species-specific bacterial and fungal communities within the *Dendrobates* genus [Figure 1; PERMANOVA, Bacteria: *p* < 0.01, *F*_(5, 45)_ = 6.98, all pair-wise *p* < 0.001; Fungi: *p* < 0.01, *F*_(5, 45)_ = 41.43, all pair-wise *p* < 0.001] and this species specificity was maintained whether the frogs were reared in separate tanks at the Animal Care Facility, or in a shared tank on exhibit. We found lower copies of the 16S rRNA gene [Figure S1; ANOVA, *p* < 0.01, *F*_(5, 45)_ = 29.01, all pair-wise *p* < 0.001] and higher ITS copies [Figure S1B, *p* < 0.01, *F*_(5, 53)_ = 63.21, all pair-wise *p* < 0.01) on frog skin compared to the environment. There was no significant difference in the number of copies of the16S rRNA gene and ITS region among the species of frogs we examined.

### Fungal isolation and *Bd* challenge assays

We isolated 135 visually distinct fungal strains, of which 90 were unique at 97% sequence identity, from the skin and environment of *Dendrobates* frogs (Table S1). The cultured isolates, when screened against the ITS amplicon sequences, accounted for 71% of the sequences present. Secondary metabolites were collected from each isolate and tested for activity against zoospores from two strains of *Bd* (JEL 197 and 423). For fungi isolated from the environment (*N* = 25), most enhanced (*N* = 12) or had no effect on (*N* = 5) *Bd* growth. Those that were inhibitory toward *Bd*, 3 could inhibit both strains of *Bd*, 3 could only inhibit JEL 423, and 3 could only inhibit JEL 197. For fungi isolated from frogs (*N* = 65), 6 had no effect on *Bd* growth. For isolates tested against *Bd* JEL 197, 19 facilitated the growth of JEL 197, while 19 inhibited the growth of JEL 197. For isolates tested against *Bd* JEL 423, 28 facilitated growth of JEL 423 and 20 inhibited the growth of JEL 423. For OTUs with more than one visually distinct isolate associated with it, they displayed similar levels of inhibition or facilitation. Phylogenetic analysis of the fungal taxa (Table S1, Figure S2) revealed diverse isolates primarily associated with phylum Ascomycota. A UniFrac significance test (Lozupone et al., 2006) indicated a significant phylogenetic signal (*p* < 0.001) for inhibition of *Bd* growth. Further, a phylogenetic signal test also indicated a significant (*p* = 0.003, *K* = 2.68) phylogenetic signal of *Bd* inhibition among fungal taxa. Together, these suggest a phylogenetic conservation of *Bd* inhibition in fungi.

### Distribution of *Bd* inhibiting/facilitating taxa

**Figure 2:**
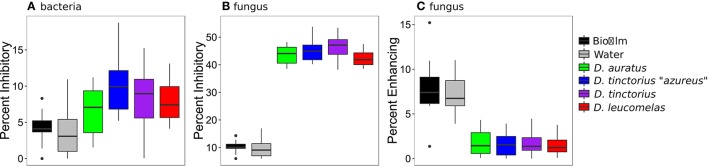
Percentage of bacterial **(A)** and fungal **(B)** communities that inhibit the growth of *Bd* and the percentage of fungal communities that facilitate the growth of *Bd* **(C)** from *Dendrobates* spp. frogs and their enclosure. Boxes represent 25–75% quartiles, and the solid black line is the median value. Note, no *Bd* facilitating bacteria were found on the frogs sampled. *Bd*-inhibitory bacteria were determined by Woodhams et al. (2015) and fungi were identified in this study.

We assessed the community composition of *Bd*-inhibitory and *Bd*-facilitating taxa by screening our high throughput sequencing data against databases of known *Bd* inhibitory/facilitating bacteria (Woodhams et al., 2015) and fungi (Table S1). Our result revealed that a significantly higher percentage of the cutaneous bacterial and fungal communities, assessed by the proportion of reads, were inhibitory than were found in the frog's environment (Figures 2A,B). However, there were significantly more *Bd* inhibitory fungi than *Bd* inhibitory bacteria [*F*_(11, 96)_ = 21.44, *p* < 0.0001]. In fact, approximately 45% of the fungal community on a given frog was capable of inhibiting *Bd*. Further, the percent of *Bd*-facilitating fungal taxa (Figure 2C) was significantly lower on frog skin than the environment [*F*_(5, 45)_ = *p* < 0.01, all pair-wise *p* < 0.01] and no *Bd*-facilitating bacteria were found on the frog skin.

**Figure 3:**
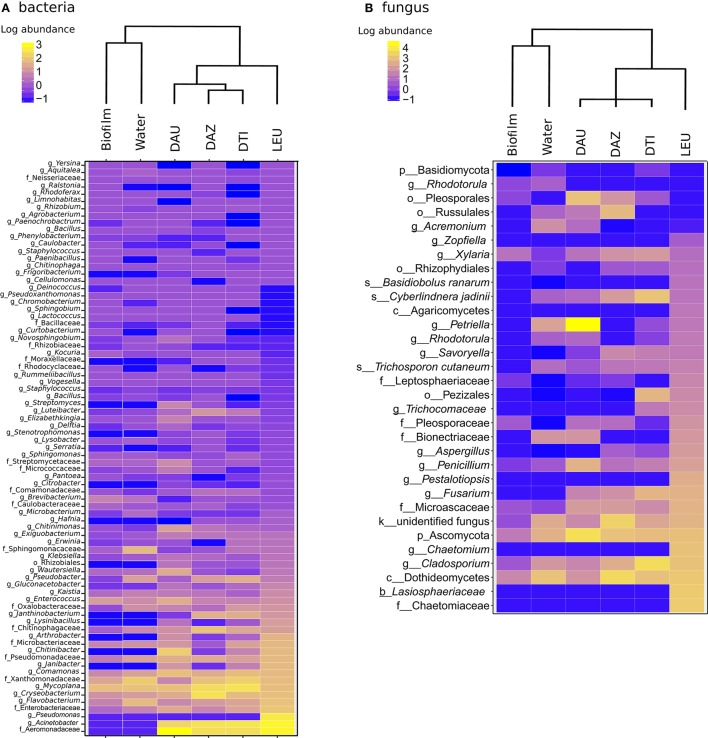
Heat maps of log_10_ abundance of *Bd*-inhibitory bacterial **(A)** and fungal **(B)** OTUs. Dendrograms are based on weighted pair group method and arithmetic mean (WPGMA) clustering. The highest level of taxonomic identification of each taxon is denoted by k, kingdom; p, phylum; o, order; f, family; and g, genus; DAU, *Dendrobates auratus;* DAZ, *D. tinctorius* “azureus”; DTI, *D. tinctorius;* LEU, *D. leucomelas*.

We next examined patterns of antifungal community structure among different *Dendrobates* species by comparing abundance and community composition of bacterial taxa that demonstrated antifungal properties. Our results indicate species specific antifungal bacterial communities and distinct anti-microbial fungal communities on the frogs relative to their environment [PERMANOVA, Bacteria- *p* < *p* < 0.001, *F*_(5, 45)_ = 12.34, all pairwise *p* < 0.01, Fungi- *p* < 0.001, *F*_(5, 45)_ = 29.43, all pairwise *p* < 0.05; Figure 3A]. Because the communities associated with *D. leucomelas* are considerably dissimilar to the other frogs, this potentially links the differential susceptibility among species of *Dendrobates* (Hirokawa et al., 2008) to their specific microbial communities (Figure 1). While many *Bd* inhibitory bacterial taxa were in similar abundance between frogs and their environment, all frogs possessed a higher proportion of bacteria from the families Aeromonadaceae, Enterobacteriaceae, Pseudomonadaceae, and Xanthomonadaceae and from the genera *Cryseobacterium, Flavobacterium*, and *Comamonas* (Figure 3A; Kruskal-Wallis test, Benjamini-Hochberg corrected *p* < 0.001). *Dendrobates leucomelas*, the species that withstood a previous *Bd* infection (Hirokawa et al., 2008), was enriched (*p* < 0.001) in bacteria from the genus *Pseudomonas*, which was in very low abundance on other frogs and in the environment. Like the bacterial communities, the *Bd* inhibitory fungal communities displayed species-specific inhibitory communities (Figure 3B). All frog species had skin containing a large number of a highly divergent fungal taxa from the phylum Ascomycota, including a taxon that was not closely related to known fungi even at the kingdom level, and from the genus *Cladosporium* (Figure 3). Compared to the other frog species, *D. leucomelas* was enriched in *Bd* inhibitory taxa from the phylum Ascomycota, in particular taxa from the families Chaetomiaceae, Lasiosphaeriaceae, and the genus *Pestalotiopsis* (*p* < 0.001), suggesting that these taxa may play a role in *Bd* defense and are potential candidates for probiotics.

**Figure 4:**
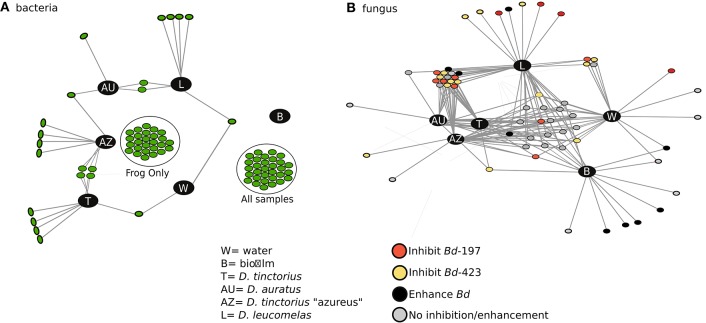
Network analysis depicting the connectivity among sample types for *Bd* inhibitory bacterial **(A)** and *Bd*-inhibitory/facilitating fungal **(B)** taxa. Each dot represents an OTU and the lines represent associations (presence) to a sample category. Different colors in **(B)** indicate the taxa's ability to either inhibit, facilitate, or have no effect on *Bd* growth. In **(A)** “Frog only” taxa are found only in frogs while “all samples” are found in all sample types, the interactions have been removed for ease of viewing. No *Bd*-facilitating bacteria were found in this study. AU, *Dendrobates auratus*; AZ, *D. tinctorius* “azureus”; T, *D. tinctorius*; L, *D. leucomelas;* W, water; B, Biofilm.

To determine the interactions between *Bd*-facilitating and -inhibiting taxa within the bacterial and fungal datasets we performed a bipartite network analysis (Figure 4), which assessed the presence/absence of taxa in different environments or frogs. In this analysis, dots represent inhibitory/facilitating OTUs and lines represent positive associations (i.e., presence) to the sample categories. Bacterial and fungal networks had similar topology, where there are several nodes (OTUs) unique to the frogs and environment. Further, there's a distinct group of OTUs shared between all sample types and OTUs shared only between frog species. Both bacterial and fungal networks indicated that frogs had a higher degree of network connectivity (associations; mean = 65.25 for bacteria, 38.75 for fungus) to *Bd*-inhibitory taxa than the environment (mean = 32 for bacteria, 28 for fungus). Further, the number of *Bd*-inhibitory taxa unique to frog skin (*n* = 47 for bacteria, *n* = 25 for fungi) was higher than those unique to the environment (*n* = 0 for bacteria, *n* = 1 for fungi) and *D. leucomelas* had the highest number of unique inhibitory taxa (*n* = 4 for bacteria, *n* = 6 for fungi). The presence of *Bd*-inhibitory bacteria and fungi on frog skin and not in the environment suggests that host factors may facilitate these microbes, while *Bd* inhibitory microbes present on both frog skin and environment may be viable candidates for use as probiotics in the treatment or prevention of chytridiomycosis.

### Probiotic stress and immune tests on midwife toads (*A. obstetricans*)

**Figure 5:**
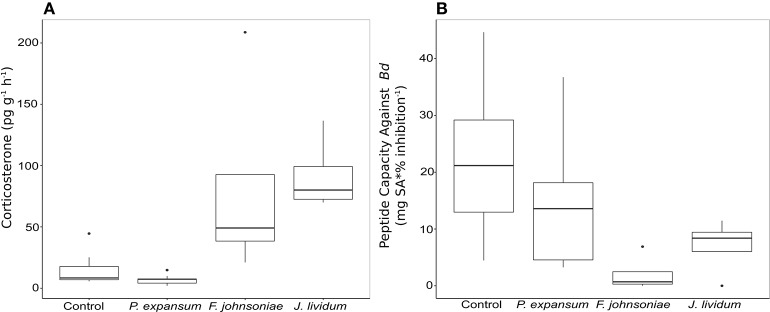
Box plot of corticosterone release rates **(A)** and toad peptide capacity against *Bd* **(B)** in Midwife Toads, *Alytes obstetricans*, measured after exposure to one fungal and two bacterial probiotic strains.

To determine potential endocrine-immune interactions produced by applications of fungal or bacterial probiotics, we exposed a non-*Bd* resistant species, Midwife toads (*A. obstetricans*), to two bacterial strains (*J. lividum* and *F. johnsoniae*) and a fungus closely related to an isolate from our data set, *P. expansum* (Table S1). These probiotics were previously isolated from the target host species. Exposure to bacterial strains significantly increased corticosterone (stress hormone) release rates relative to *P. expansum* and control frogs (Figure 5A, Figure S3; ANOVA, *F* = 21.83, *p* < 0.001). Exposure to each bacterial strains decreased host antimicrobial peptide capacity against *Bd* (Figure 5B, Figure S3; ANOVA, *F* = 4.26, *p* = 0.015), while exposure to *P. expansum* did not significantly alter peptide activity relative to control frogs.

## Discussion

Host-associated microbial communities can have profound effects on host health and immune response (Cho and Blaser, 2012). While microbes can directly modulate the host immune function through direct interactions with the host (i.e., inflammation), host associated taxa can provide additional immune defense against pathogens through the production of secondary metabolites. Our study examined the role of host-associated bacterial and fungal communities in immune defense against chytridiomycosis in three species of poison arrow frogs (*Dendrobates* spp.). We observed species-specific bacterial and fungal communities associated with each frog that was distinct from their enclosure. Species-specific microbial communities have been observed at broad (Givens et al., 2015), narrow (Lee et al., 2011), and sub-species levels (Micallef et al., 2009). Previous work on fungi in other host-associated systems has documented similar findings to ours (e.g., Porras-Alfaro and Bayman, 2011; Bálint et al., 2015), however, our work builds upon previous work by linking host-environmental differences to the presence of beneficial, anti-fungal taxa. Further, the differences in microbiota observed among different frog species held within the same exhibit, as well as the consistency in community structure sampled in two different years, suggest that these frogs may recruit and maintain specific taxa, including those that may provide protection against *Bd*. Our study did not include field-caught frogs and future studies of wild frogs will help elucidate the strength of the pattern.

Much effort has been spent identifying bacteria capable of inhibiting *Bd* to be used as probiotics (Bletz et al., 2015; Woodhams et al., 2015; Walke and Belden, 2016) and some bacteria have been shown to facilitate the growth of *Bd* zoospores (Woodhams et al., 2015). The role of fungal taxa in inhibiting *Bd*, in contrast, has been understudied. Our study isolated 135 previously uncultivated fungi from amphibians, 44 of which inhibited the growth of *Bd* zoospores. Further, the proportion of the fungal community inhibitory toward *Bd* was significantly higher than the proportion of bacteria on frog skin, supporting our hypothesis that fungi contribute more to host defense than bacteria. Further, it suggests fungi have the potential to be an important source of host defense against pathogens on amphibians and perhaps for other organisms (Dean et al., 2012). The higher proportion of *Bd*-inhibitory fungi, while not an explicit test of the efficacy of fungi to supplement host immunity, suggests that there is the potential of fungi to aid in host defense. These data support our second hypothesis, however, an explicit test of fungal probiotics in *Bd* infected amphibians is needed to fully elucidate the role of fungi in the amphibian immune response.

In addition to fungal taxa with the ability to inhibit the growth of *Bd* zoospores, we identified several (*n* = 20 from environment, 19 from frog) taxa capable of facilitating the growth of *Bd* zoospores. The taxa that could facilitate the growth of *Bd* were predominately from the phylum Basidiomycota, a phylum distantly related to the Chytridiomycota phylum (Choi and Kim, 2017). While cooperation between pathogens and microbes is more common between closely related taxa (West and Buckling, 2003; Griffin et al., 2004), our results suggest that cooperation between fungal taxa may occur across greater phylogenetic distance, indicating a lack of specificity in these interactions. Further, the presence of *Bd*-inhibiting and *Bd*-facilitating fungal taxa suggests that the interactions between skin-associated taxa and potential probiotics is important for not only the establishment of potential probiotics (Becker et al., 2011; Küng et al., 2014; Kueneman et al., 2016) but also for the immunological function of host-associated microbial communities.

Frogs from the *Dendrobates* population at the New England Aquarium have demonstrated differential susceptibility to *Bd* infection, with *D. leucomelas* having previously demonstrated the ability to clear *Bd* infection (Hirokawa et al., 2008). Analysis of bacterial and fungal communities revealed species-specific fungal and bacterial communities, suggesting the microbial communities may play an important role in host defense, in particular for *D. leucomelas*. Further, network analysis revealed groups of *Bd*-inhibitory bacteria and fungi found only on the skin of *D. leucomelas*, suggesting these taxa may play a role in the ability of *D. leucomelas* to clear *Bd* infection. *Bd* inhibiting taxa from the genus *Pseudomonas* were enriched on the microbiome of *D. leucomelas* relative to other frogs and taxa from this genus was absent in other species and the environment. The genus *Pseudomonas*, a common bacterial genus across many biomes, is known to produce numerous extracellular and often antimicrobial metabolites (Holmström and Kjelleberg, 1990) and its use as a probiotic has proven effective in plants (Picard and Bosco, 2008), fish (El-Rhman et al., 2009), and shellfish (Van Hai and Fotedar, 2009). In addition to bacteria, *D. leucomelas* was enriched in *Bd*-inhibiting fungi from the families Chaetomiaceae, Lasiosphaeriaceae, and the genus *Pestalotiopsis*, suggesting a potential role for these taxa in *Bd* defense. The persistence of *Bd*-inhibiting taxa on amphibian skin, as well cosmopolitan distribution of many of these taxa across biomes suggests they could be potential probiotic candidates for treating chytridiomycosis.

Our results demonstrated a potential link between amphibian-associated fungi and host defense against *Bd* infection. While we were limited to a small study using the endangered midwife toad, probiotic application of *P. expansum* to midwife toads did not significantly alter host immune or stress levels, while bacterial probiotics did. Applications of the probiotic bacterium *J. lividum* were protective against chytridiomycosis for several species of amphibians (Becker et al., 2009; Harris et al., 2009; Kueneman et al., 2016). Further, the viability of *Bd* zoospores was significantly reduced after exposure to mucus from frogs treated with the bacterium *F. johnsoniae* and the fungus *P. expansum* (Woodhams et al., 2014). However, a recurrent problem with probiotic applications is the colonization resistance of hosts (Becker et al., 2011; Küng et al., 2014), such that augmented bacteria fail to establish in the skin, particularly in the absence of an environmental reservoir for the probiotic bacteria (Kueneman et al., 2016). Additionally, Küng et al. (2014) showed that some probiotic treatments may stress hosts or cause an immune reaction in amphibians that prevents establishment of the probiotic. Our results suggest that fungal taxa such as *Penicillium*, that are common across amphibians and environments, may be potential candidates for amphibian probiotic therapy, as has been previously shown in agricultural systems (Fox, 2015). Fungal probiotics may not induce a host stress responses or repress the host's mucosal peptide response, although the generality of this finding among hosts and potential probiotics is not known. Instead, fungal probiotics may produce antimicrobial metabolites while inducing host defenses that target foreign fungi, including *Bd*.

## Conclusions

We show species-specific bacterial and fungal communities associated with *Dendrobates* frogs that are distinct from their environment. The distinct microbiome (including the mycobiome) on host skin is, in part, due to host recruitment of potentially anti-microbial taxa that may help promote host health. Frog-associated bacterial communities possessed a significant portion of *Bd*-inhibitory taxa and the fungal communities were dominated by *Bd* inhibitory taxa, suggesting fungi may play a greater role in host protection than bacteria in amphibians. Our results suggest that host-associated fungi should be a greater focus of future efforts to develop probiotic therapies for the treatment of chytridiomycosis and attempts at manipulation and probiotic experiments are needed to fully elucidate this relationship. When considering the host immune priming function provided by microbiota (Kurtz and Scharsack, 2007), and host resistance to bacterial colonization (Küng et al., 2014), fungi may provide key probiotics needed for disease management in amphibians.

## Data availability

Fungal isolate sequences can be found in NCBI under accession numbers KY114967-KY115101. High-throughput sequencing data can be found in the NCBI Sequence Read Archive under accession numbers PRJNA354614 and PRJNA354619.

## Author contributions

PK, JLB, MT, and DW designed the poison arrow frog experiment. PK oversaw the students that performed all sequencing related activities. SF and PK performed the qPCR, *Bd* inhibition assays, and isolation of fungal cultures. SF-B, CG, JB, and DW performed midwife toad experiments. PK performed all sequence and statistical analyses. PK, JLB, MT, and DW wrote the paper with contributions from SF, SF-B, CG, and JB.

### Conflict of interest statement

The authors declare that the research was conducted in the absence of any commercial or financial relationships that could be construed as a potential conflict of interest.
