# Supplementary material for: Fight Fungi with Fungi: Antifungal Properties of the Amphibian Mycobiome
Source: Front Microbiol. 2017 Dec 14;8:2494. doi: 10.3389/fmicb.2017.02494 (PMC5735112; doi:10.3389/fmicb.2017.02494)
Supplement: Supplementary file 1 [file DataSheet1.docx]

**Supplementary Figures and Tables**

Figure S1- Quantitative PCR results comparing the abundance of bacteria (A) and fungus (B) in *Dendrobates* spp. frogs and their enclosure. Boxes represent 25-75% quartiles and the solid black line is the median value. Note the differing scales between (A) and (B).

Figure S2- Ultrametric phylogenetic tree of distinct fungal isolates (at 97% sequence identity) based on maximum likelihood. OTUs are colored by the ability to inhibit (blue), enhance (red), or have no significant effect (black) on Bd growth. All branches colored red have bootstrap values >0.75, green branches have bootstrap values >0.5, and black branches have bootstrap values <0.50. Sequences from *Bd* and the salamander chytrid pathogen (*B. salamamdrivorans*) to show the relationship to the amphibian pathogens.

Figure S3- Plot of corticosterone levels versus peptide capacity against *B. dendrobatidis* (*Bd*) for Midwife Toads, *Alytes obstetricans*, exposed to three potential probiotic microbes or no exogenous microbes.


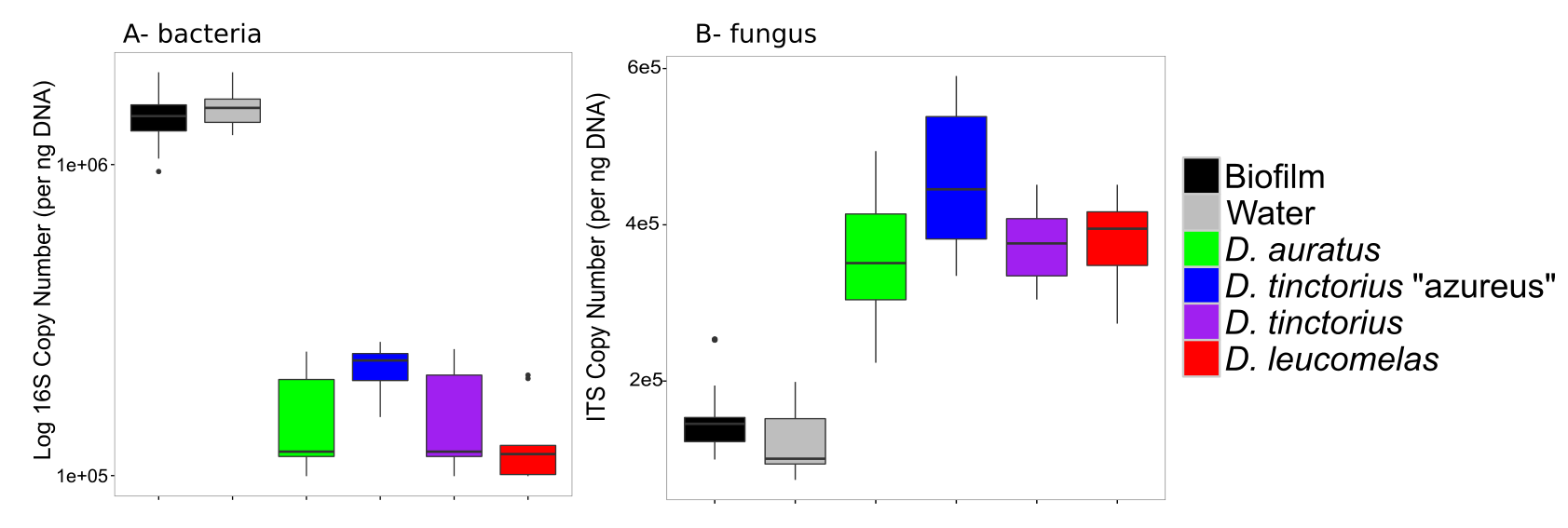


Figure S1.


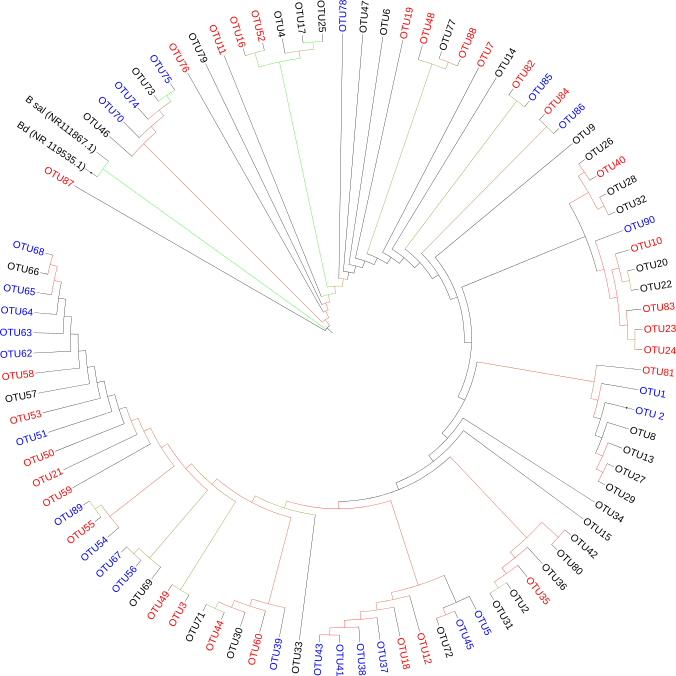


Figure S2

Figure S3
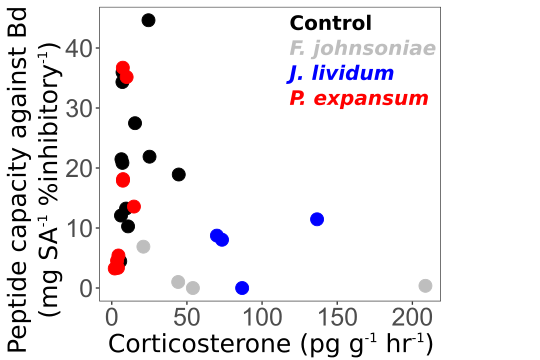


Table S1- List of unique fungal isolates (at 97% sequence identity) and whether or not they possess the ability to significantly (Benjamini Hochberg *p*<0.05) inhibit (<0%) or enhance (>0%) the growth of two strains of *Bd,* strains 197 and 423. Numbers in parentheses are standard error of the mean. The highest level of taxonomic classification, as determined by BLASTn is indicated as is the accession number in NCBI. p= phylum, c=class, o=order, f=family, g=genus, s=species, and ns= not significant.

| OTU | Taxonomy | Inhib. 197 | Inhib. 423 | Isolation Source | NCBI Accession |
| --- | --- | --- | --- | --- | --- |
| 1 | p_Ascomycota | -50.68 (22.92) | -33.01 (5.85) | D. leucomelas | KY114967 |
| 2 | p_Ascomycota | ns | -50.43 (-10.58) | Tank Water | KY114968 |
| 3 | c__Dothideomycetes | ns | 72.84 (-22.68) | D. tinctorius "azureus" | KY114969 |
| 3 | c__Dothideomycetes | 81.45 (2.61) | ns | D. leucomelas | KY114970 |
| 4 | c__Dothideomycetes | ns | 47.35 (-29.33) | Tank Water | KY114971 |
| 5 | c__Dothideomycetes | ns | 43.02 (-19.88) | D. auratus | KY114972 |
| 6 | c__Dothideomycetes | ns | ns | D. tinctorius "azureus" | KY114973 |
| 7 | c__Dothideomycetes | 71 (8.59) | 54.87 (49.98) | D. tinctorius "azureus" | KY114974 |
| 8 | c__Dothideomycetes | 69.85 (7.83) | ns | D. leucomelas | KY114975 |
| 9 | c__Dothideomycetes | 66.26 (23.05) | ns | D. leucomelas | KY114976 |
| 10 | c__Dothideomycetes | 68.1 (4.95) | 132.33 (19.46) | Biofilm | KY114977 |
| 11 | c__Dothideomycetes | 65.99 (19.81) | 45.12 (6.7) | Biofilm | KY114978 |
| 12 | c__Dothideomycetes | 74.15 (16.71) | 95.39 (5.18) | D. tinctorius "azureus" | KY114979 |
| 13 | c__Dothideomycetes | 40.3 (45.41) | 41.51 (11.06) | D. tinctorius "azureus" | KY114980 |
| 14 | c__Dothideomycetes | ns | 58.63 (-7.64) | Biofilm | KY114981 |
| 15 | c__Dothideomycetes | 85 (15.18) | 57.86 (2.53) | D. tinctorius "azureus" | KY114982 |
| 16 | c__Dothideomycetes | 86.62 (11.47) | 65.49 (12.2) | Biofilm | KY114983 |
| 17 | c__Dothideomycetes | ns | 42.02 (-2.48) | D. auratus | KY114984 |
| 18 | c__Dothideomycetes | 40.6 (40.07) | 49.67 (15.84) | Biofilm | KY114985 |
| 19 | c__Dothideomycetes | 61 (46.52) | 52.5 (6.17) | D. tinctorius "azureus" | KY114986 |
| 20 | c__Dothideomycetes | 67.92 (11.98) | ns | D. tinctorius "azureus" | KY114987 |
| 21 | c__Dothideomycetes | 85.96 (3.07) | 89.84 (3.08) | D. auratus | KY114988 |
| 22 | c__Dothideomycetes | ns | 54.72 (-7.59) | D. auratus | KY114989 |
| 23 | c__Dothideomycetes | ns | 87.96 (-8.07) | D. leucomelas | KY114990 |
| 24 | c__Dothideomycetes | 40.99 (14.38) | 66.61 (5.91) | D. leucomelas | KY114991 |
| 25 | c__Dothideomycetes | 63.04 (7.98) | ns | Tank Water | KY114992 |
| 26 | c__Dothideomycetes | ns | 87.85 (-13.02) | Biofilm | KY114993 |
| 27 | c__Dothideomycetes | ns | ns | Biofilm | KY114994 |
| 28 | c__Dothideomycetes | ns | 61.41 (-10.54) | Tank Water | KY114995 |
| 29 | c__Dothideomycetes | -98.22 (2.28) | -85.06 (6.04) | D. leucomelas | KY114996 |
| 30 | c__Dothideomycetes | ns | ns | Biofilm | KY114997 |
| 31 | c__Dothideomycetes | 43.23 (22.71) | ns | D. auratus | KY114998 |
| 32 | c__Dothideomycetes | 111.57 (11.5) | ns | D. leucomelas | KY114999 |
| 33 | g__Cladosporium | ns | ns | D. auratus | KY115000 |
| 34 | g__Arthrographis | ns | 39.53 (-25.53) | D. auratus | KY115001 |
| 35 | o__Pleosporales | ns | 81.82 (-2.83) | D. auratus | KY115002 |
| 36 | o__Pleosporales | -33.43 (7.8) | -101.77 (0.38) | D. auratus | KY115003 |
| 37 | f__Leptosphaeriaceae | -46.26 (10.03) | -25.8 (5.22) | Tank Water | KY115004 |
| 38 | f__Phaeosphaeriaceae | 2.37 (9.2) | 118.14 (16.98) | D. auratus | KY115005 |
| 39 | f__Pleosporaceae | -81.22 (7.27) | -100.05 (0.33) | D. leucomelas | KY115006 |
| 40 | g__Curvularia | 73.03 (11.82) | 49.08 (57.17) | Tank Water | KY115007 |
| 41 | g__Aspergillus | -35.79 (13.09) | -41.79 (6.59) | D. auratus | KY115008 |
| 42 | g__Penicillium | -82.29 (3.88) | -78.2 (4.73) | D. tinctorius "azureus" | KY115009 |
| 43 | s__Trichocomaceae sp | -60.03 (6.37) | -51.86 (12.47) | D. leucomelas | KY115010 |
| 44 | f__Teloschistaceae | ns | 84.71 (-18.47) | D. auratus | KY115011 |
| 45 | o__Pezizales | -42.51 (10.44) | -40.7 (9.16) | D. auratus | KY115012 |
| 46 | s__Helvella sp | 37.91 (26.38) | ns | Biofilm | KY115013 |
| 47 | s__Plectania milleri | ns | ns | Tank Water | KY115014 |
| 48 | s__Cyberlindnera jadinii | -80.97 (7.66) | -53.15 (9.42) | D. tinctorius "azureus" | KY115015 |
| 49 | c__Sordariomycetes | ns | 46.54 (-14.42) | D. auratus | KY115016 |
| 50 | o__Hypocreales | 83.13 (6.32) | ns | D. leucomelas | KY115017 |
| 51 | f__Bionectriaceae | -18.6 (44.96) | -89.67 (10.76) | D. leucomelas | KY115018 |
| 52 | f__Hypocreaceae | 56.38 (10.55) | 43.6 (37.45) | D. leucomelas | KY115019 |
| 53 | s__Trichoderma spirale | 61.67 (44.03) | 63.16 (43.33) | D. leucomelas | KY115020 |
| 54 | s__Acremonium sp | -84.46 (5.21) | -24.39 (7.69) | D. leucomelas | KY115021 |
| 55 | g__Sarocladium | 39.34 (32.12) | 46.8 (15.26) | D. tinctorius "azureus" | KY115022 |
| 56 | g__Fusarium | -27.6 (8.33) | -63.17 (13.08) | D. leucomelas | KY115023 |
| 57 | s__Nectriaceae sp | ns | ns | D. auratus | KY115024 |
| 58 | s__Volutella consors | 51.4 (3.5) | 71.24 (27.13) | D. tinctorius "azureus" | KY115025 |
| 59 | f__Ophiocordycipitaceae | 68.38 (11.05) | 51.43 (34.1) | D. tinctorius "azureus" | KY115026 |
| 60 | s__Purpureocillium lilacinum | ns | 50.86 (-32.07) | D. tinctorius "azureus" | KY115027 |
| 62 | g__Savoryella | -71.51 (9.79) | 22.17 (13.55) | D. leucomelas | KY115028 |
| 63 | f__Microascaceae | -38.85 (13.72) | -128.22 (26.7) | D. auratus | KY115029 |
| 64 | f__Chaetomiaceae | -68.38 (28.96) | -57.47 (16.08) | Biofilm | KY115030 |
| 65 | g__Chaetomium | -40.14 (6.9) | -55.13 (15.71) | Tank Water | KY115031 |
| 66 | s__Lasiosphaeriaceae sp | ns | ns | D. tinctorius "azureus" | KY115032 |
| 67 | s__Lasiosphaeriaceae sp | -68.83 (14.94) | -44.39 (12.91) | D. tinctorius "azureus" | KY115033 |
| 68 | s__Zopfiella sp | -66.32 (1.43) | -48.71 (4.99) | D. auratus | KY115034 |
| 69 | s__Zopfiella sp | ns | ns | D. tinctorius "azureus" | KY115035 |
| 70 | g__Pestalotiopsis | ns | -27.61 (-9.25) | D. auratus | KY115036 |
| 71 | s__Microdochium sp | ns | ns | D. leucomelas | KY115037 |
| 72 | g__Xylaria | -18.01 (6.66) | 182.61 (54.06) | D. tinctorius "azureus" | KY115038 |
| 73 | p__Basidiomycota | -31.54 (7.29) | 30.6 (4.66) | D. tinctorius "azureus" | KY115039 |
| 74 | p__Basidiomycota | -78.96 (0.34) | 67.32 (15.56) | Tank Water | KY115040 |
| 75 | c__Agaricomycetes | -32.44 (7.02) | ns | Tank Water | KY115041 |
| 76 | o__Agaricales | ns | 62.87 (-17.88) | D. auratus | KY115042 |
| 77 | f__Phanerochaetaceae | ns | ns | Biofilm | KY115043 |
| 78 | o__Russulales | -35.38 (32.78) | ns | Tank Water | KY115044 |
| 79 | g__Lactarius | ns | ns | Biofilm | KY115045 |
| 80 | g__Stereum | 34.58 (12.45) | 208.51 (22.9) | D. auratus | KY115046 |
| 81 | s__Sebacinales Group B sp | 44.13 (15.26) | ns | D. auratus | KY115047 |
| 82 | c__Microbotryomycetes | 44.91 (3.9) | 39.33 (38.43) | D. auratus | KY115048 |
| 83 | g__Rhodotorula | 119.74 (57.49) | ns | D. auratus | KY115049 |
| 84 | s__Rhodotorula mucilaginosa | ns | 88.14 (-53.86) | Tank Water | KY115050 |
| 85 | g__Rhodotorula | ns | -55.9 (-45.79) | D. leucomelas | KY115051 |
| 86 | s__Trichosporon cutaneum | ns | -22.2 (-13.56) | D. tinctorius "azureus" | KY115052 |
| 87 | o__Rhizophydiales | 64.27 (11.81) | 64.59 (5.6) | Tank Water | KY115053 |
| 88 | s__Rozellomycota sp | 37.79 (25.27) | 75.55 (24.48) | D. auratus | KY115054 |
| 89 | k__unidentified fungus | ns | -99.86 (-0.89) | D. auratus | KY115055 |
| 90 | s__Basidiobolus ranarum | -62.83 (21.83) | -75.7 (6.72) | D. leucomelas | KY115056 |
